# Cricopharyngeal myotomy using grasping scissors: Benefits of peroral endoscopic myotomy in symptomatic Zenker’s diverticulum

**DOI:** 10.1055/a-2703-3219

**Published:** 2025-10-13

**Authors:** Marc Harb, Jean Baptiste Danset, Cynthia Medlij, Olivier Marty, Damien Levoir, Elise Chanteloup, Bernard El Khoury, Christophe Souaid, Yann Le Baleur

**Affiliations:** 1 55662 Gastroenterology Unit, Fondation Hopital Saint Joseph, Paris, France

**Keywords:** Endoscopy Upper GI Tract, Motility / achalasia, POEM, GI Pathology

## Abstract

**Background and study aims:**

Multiple therapeutic modalities, including surgery and rigid and flexible endoscopy, have been adopted to manage Zenker’s diverticulum (ZD). Relief from symptoms such as dysphagia and regurgitation is the main goal of therapy in symptomatic ZD. This study was the first large cohort that aimed to assess efficacy with time and safety of endoscopic diverticulotomy using the Clutch Cutter.

**Patients and methods:**

Cricopharyngeal myotomy was performed in 43 patients at Hospital Saint Joseph de Paris, a tertiary referral center. Symptoms were analyzed before and at 3, 6, and 12 months post-intervention using an extensive questionnaire about dysphagia, odynophagia, regurgitation, chronic cough, state of health, and complications. Procedure details such as duration, complications, and technical success were recorded.

**Results:**

Mean size of ZD was 25.6 mm. Mean procedure time was 48 minutes. No major complications (e.g., perforation, mediastinitis) occurred, although one patient suffered from a sinus piriform wound. Follow-up was performed at 3, 6, and 12 months. During follow-up consultations, patients rated improvement in their symptoms as a percentage. At 12 months, 97% of patients reported 100% improvement.

**Conclusions:**

In patients with treatment-naïve ZD, the Clutch Cutter technique is safe, fast, and provides durable symptom remission.

## Introduction

Zenker's diverticulum (ZD) stands out as the most well-known among pharyngoesophageal diverticula. It presents itself as a hernia involving the mucosal and adventitial layers, typically forming within Killian's triangle, a weak area located between the cricopharyngeal muscle and the thyropharyngeal muscle. This condition is relatively rare, with an estimated overall prevalence ranging from 0.01% to 0.11%. It predominantly affects men in their seventh and eighth decades of life 1 .

ZD can be asymptomatic, but individuals may experience symptoms such as dysphagia, regurgitation, and associated complications. Complications may include aspiration pneumonia, often linked to regurgitation of food into the respiratory tract, sometimes occurring recurrently. In cases of prolonged dysphagia, malnutrition can develop as a severe complication.

Diagnosis typically relies on fluoroscopic evaluation, which is the examination recommended by the European Society of Gastrointestinal Endoscopy (ESGE) 2 3 . However, diagnosis can also be confirmed through endoscopy and cross-sectional imaging.

Treatment is generally reserved for symptomatic ZD. The goal of treatment aligns with the underlying pathophysiology, aiming to restore complete opening of the upper esophageal sphincter by cutting the cricopharyngeal muscle. This intervention helps prevent intrabolus hyperpressure in the hypopharyngeal region, subsequently alleviating dysphagia and its associated issues.

Currently, therapeutic options primarily include external surgery through cervicotomy (extramucosal myotomy), but indications for this approach are increasingly limited due to more frequent adverse events (AEs) and morbidity than alternative endoscopic treatments. Alternatively, either rigid endoscopic approach or interventional flexible endoscopy (transmucosal myotomy) are available.

The endoscopic approach has become the preferred choice for treatment due to its advantages, such as shorter operative times, quicker return to eating, shorter hospitalization periods, and a lower complication rate compared with the traditional cervical approach. This preference holds true except in patients who exclusively require extramucosal myotomy 4 5 6 .

ESGE suggests that when conducting endoscopic septotomy, it is advisable to carry out a thorough myotomy of the cricopharyngeal muscle. Every expert endoscopist engaged in management of ZD through flexible endoscopy strongly emphasizes the necessity for a full transection of the cricopharyngeus muscle.

Treatment by flexible endoscopy, including procedures like septotomy and Zenker’s peroral endoscopic myotomy (Z-POEM), plays a crucial role in managing the treatment of ZD.

Septotomy is considered a safe and effective treatment for ZD. It is less invasive than traditional surgical approaches, with shorter recovery times. During septotomy, an endoscope is inserted through the mouth to access the diverticulum. The septum, or wall, between the esophagus and the diverticulum is then divided or incised, creating an opening. This allows food and liquids to pass through more easily, reducing symptoms associated with ZD.

During Z-POEM, an endoscope is inserted through the mouth and into the esophagus, and a submucosal cushion is injected into the submucosal space, creating a submucosal bleb. Incision of the mucosa and submucosa is conducted with an endoscopic submucosal dissection (ESD) knife to create a sufficient space to insert the tip of the scope, and submucosal dissection is used to create a tunnel. Submucosal dissection is extended until the cricopharyngeal muscle is exposed, and complete transection of the muscle is performed. Mucosal entry is closed through the scope using an endoclip after complete cricopharyngeal muscle transection to avoid any leakage. The theoretical advantage of Z-POEM is to allow complete transection of the cricopharyngeal muscle, which tends to reduce risk of recurrence.

Complications, especially infectious ones, tend to be more frequent compared with classic septotomy, and the diverticulum pouch remains intact with the risk of poor amelioration of retention symptoms, such as food or liquid regurgitation or aspiration pneumonia. For these reasons, ESGE guidelines do not recommend Z-POEM as the first endoscopic approach for symptomatic ZD.

Septotomy can be performed using various tools, including Zimmon's needle, ESD knife, and dissecting scissors. Conventionally, the incision typically stops about 3 to 5 mm before reaching the bottom of the diverticulum to avoid perforation, but this practice potentially leaves some muscle fibers intact, which can be a source of recurrence.

In theory, the advantage of using scissors is the ability to provide traction to the muscle fibers rather than making an incision by pushing. This muscle fiber traction approach may reduce risk of perforation and limit risk of recurrences, considering the complete section of the cricopharyngeal muscle fibers.

This report introduces a novel application of the Clutch Cutter (CC) device for flexible endoscopy. Originally designed for ESD, the Clutch Cutter (CC; FUJIFILM Corporation, Tokyo, Japan) is a forceps-type resection device capable of simultaneously grasping, cutting, or coagulating tissue using concentrated electrosurgical current at the closed blade, thereby preventing unintended incisions 7 8 .

## Patients and methods

This was a retrospective, single-center cohort study including 43 patients over 18 years of age with symptomatic ZD. All patients treated endoscopically using the Clutch Cutter technique for the diverticulum in the digestive endoscopy unit of Saint Joseph Hospital in Paris between 2018 and 2023 were included in this study.

All patients received both oral and written information.

Data collected were: age, sex, body mass index (BMI), symptoms related to ZD before and after the procedure, duration of hospitalization after the procedure, and the duration of the intervention.

The following data were collected from the patients: dysphagia according to the Dakkak score (0 = absence of dysphagia, 1 = dysphagia to solids, 2 = dysphagia to semi-solids, 3 = dysphagia to liquids, 4 = aphagia); other symptoms related to the diverticulum (regurgitation, cough, hypersialorrhea); and complications of the procedure. The follow-up of all patients during each period was carried out through in-person clinical consultations to evaluate the improvement of symptoms in the short (1–3 months), medium (6 months), long term (> 1 year) and even up to 2 years in some patients. Table 1 summarizes study parameters and characteristics of the patients included in it.

**Table:** **Table 1** Summary of study parameters and patient characteristics.

**Category**	**Details**
Study type	Retrospective, single-center cohort study
Patient group	43 patients over 18 years with symptomatic Zenker's diverticulum
Treatment method	Endoscopic treatment using the Clutch Cutter technique
Location	Saint Joseph Hospital, Paris
Study period	2018–2023
Patient information	All patients received oral and written information
Collected data	Age, sex, BMI, symptoms before and after the procedure, hospitalization duration, procedure duration
Dakkak score	0 = no dysphagia, 1 = dysphagia to solids, 2 = dysphagia to semi-solids, 3 = dysphagia to liquids, 4 = aphagia
Other symptoms	Regurgitation, cough, hypersialorrhea, complications of the procedure
Follow-up periods	Short-term (1–3 months), medium-term (6 months), long-term (>1 year), up to 2 years for some patients
Follow-up method	In-person clinical consultations
Main objective	Symptom improvement evaluation; no radiological or endoscopic follow-up after the procedure
BMI, body mass index.	

We were also able to perform follow-up on a group of patients for up to 2 years.

Follow-up of symptom improvement was our main objective, because from the beginning, we only treated symptomatic patients and not those based on imaging. Therefore, no radiological or endoscopic follow-up was performed after the procedure.

### Myotomy of the cricopharyngeal muscle with the Clutch Cutter

All endoscopic procedures were performed under general anesthesia with orotracheal intubation, and the patient was in a supine position. A standard gastroscope (Fujifilm EG-760R) with a short distal transparent hood (Olympus D-201–10704) was used for the procedure.

General anesthesia is the protocol established by the anesthesia department for long procedures with a risk of bleeding or complications such as perforation. In our center, general anesthesia is systematically used to ensure optimal airway management and maximum safety throughout the procedure. It also allows for better control of patient immobility, which is essential when using instruments such as the Clutch Cutter.

An upper gastrointestinal endoscopy was first performed to eliminate any contraindications, such as severe esophagitis or upper esophageal stenosis associated with the ZD. Depth of the diverticulum was estimated using a 10-mm polypectomy snare. Any liquid or food remnants in the pouch were aspirated or captured with rat-tooth forceps.

The technique used for septotomy was a modified version of the window technique previously described by Calavas et al. 9 .

Incision of the mucosa and submucosa was performed in two areas at the edge of the septum with the Clutch Cutter, using the Endocut I mode, effect 2 (2–2) VIO 3 Erbe. After creating a mucosal and submucosal incision, the space between the two incisions was snared with a 10-mm polypectomy snare (Olympus Snare SD-990–10), and mucosa and submucosa were removed in the same manner as a polypectomy using Endocut Q mode, effect 2 (2–2), with exposure of the cricopharyngeal muscle fibers. Sectioning of the cricopharyngeal fibers was performed in an anterograde manner by grasping and pulling the muscle with the Clutch Cutter using Endocut I mode, effect 2 (2–2) VIO 3 Erbe. Muscle fiber sectioning was extended until the bottom of the diverticulum pouch was reached. Hemostasis of intraprocedural bleeding or prophylactic hemostasis of visible vessels was performed with the Clutch Cutter in coagulation mode (Erbe VIO 3 Forced Coag 3.5).

Complete closure of the defect was performed using through-the-scope clips. Amoxicillin/clavulanic acid 2 g was given during the procedure and continued for 3 days (1g * 3 per day).

**Fig. 1:**
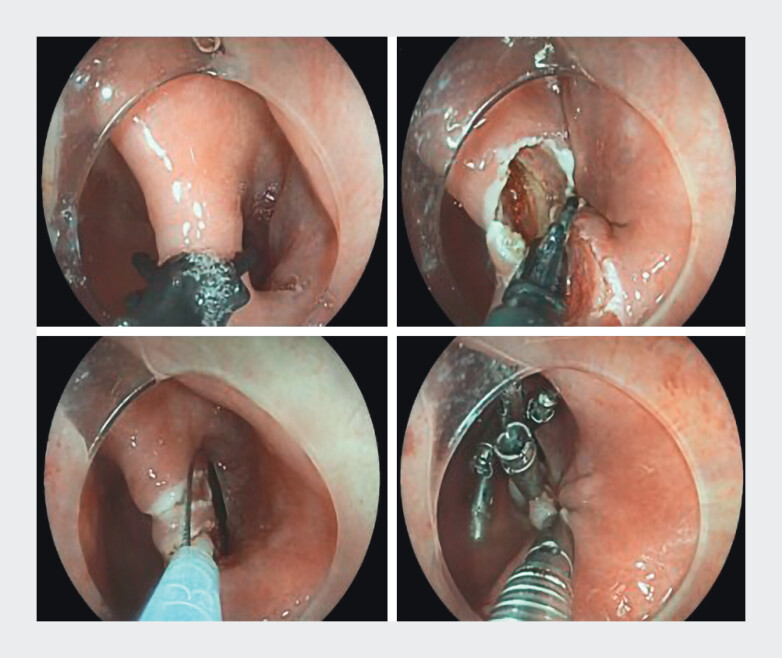
Steps in the Clutch Cutter technique.

Patients were kept NPO (nothing by mouth) the night following the treatment, and in the absence of complications, a clear liquid diet was authorized in the morning, followed by a smooth regimen for 10 days. Fig. 1 shows the steps of the Clutch Cutter technique.

## Results

In this study, 43 patients were included (26 men, 60.5%, and 17 women, 39.5%) with a mean age of 75 years (range: 51–94) and a BMI of 22.64 kg/m ^2^ .

Dysphagia was the most common symptom at consultation, present in 34 of 43 patients (79.1%). The symptom was assessed using the Dakkak score, revealing the following distribution: Score 0 (6/43, 14%), 1 (28/43, 65.1%), 2 (1/43, 2.3%), 3 (7/43, 16.3%), and 4 (1/43, 2.3%).

Other prevalent symptoms included regurgitation (30/43, 69.76%), cough (8/43, 18.6%), hypersialorrhea (8/43, 18.6%), nocturnal symptoms (5/43, 11.6%), weight loss (5/43, 11.6%), and pneumonia (3/43, 6.97%).

Pharyngo-esophageal transit was conducted in 29 of 43 patients (67.4%) during the diagnostic assessment, whereas 30 of 43 patients (69.8%) underwent esophagogastroduodenoscopy.

Mean diverticulum size, measured as the height in millimeters, was 25.6 mm between the bottom of the pocket and the collar on the lateral image of the pharyngoesophageal transit.

Among the 43 patients, seven (16.3%) had a ZD < 2 cm, 31 (72.1%) had a diverticulum ranging from 2 to 4 cm, and five (11.6%) had a diverticulum > 4 cm, reflecting a variety of sizes in the sample.

Average procedure time for diverticulotomy with cricopharyngeal myotomy was 48 minutes (range: 25–82 minutes), and mean hospital stay was approximately 2 days (range: 2–3 days). Table 2 summarizes procedure outcomes and associated data.

**Table:** **Table 2** Summary of procedure outcomes and associated data.

Parameter	Number of patients/value	Percentage/range
Pharyngo-esophageal transit conducted	29/43	67.4%
Esophagogastroduodenoscopy performed	30/43	69.8%
Mean diverticulum size (mm)	25.6 mm	-
Average procedure time (minutes)	48 min	25–82 min
Mean hospital stay (days)	~2 days	2–3 days

**Fig. 2:**
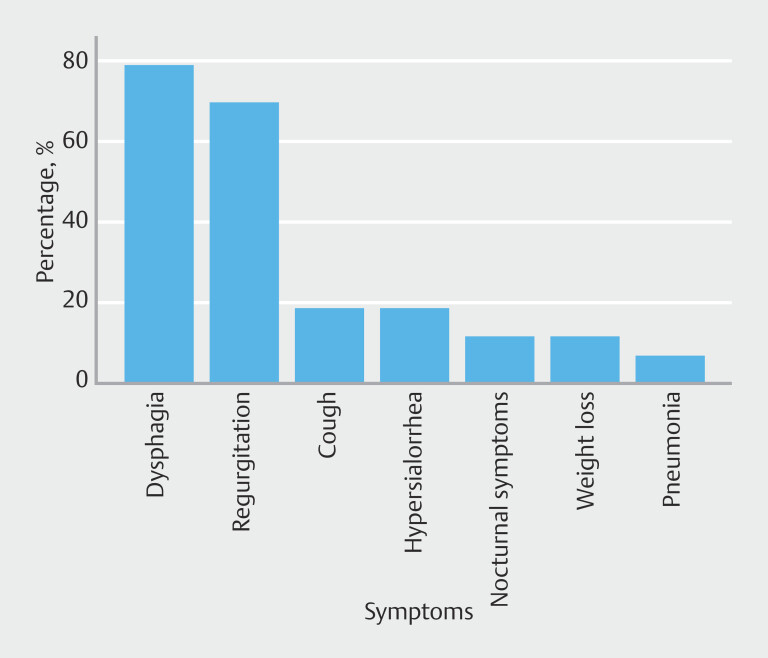
Prevalence of symptoms.

**Fig. 3:**
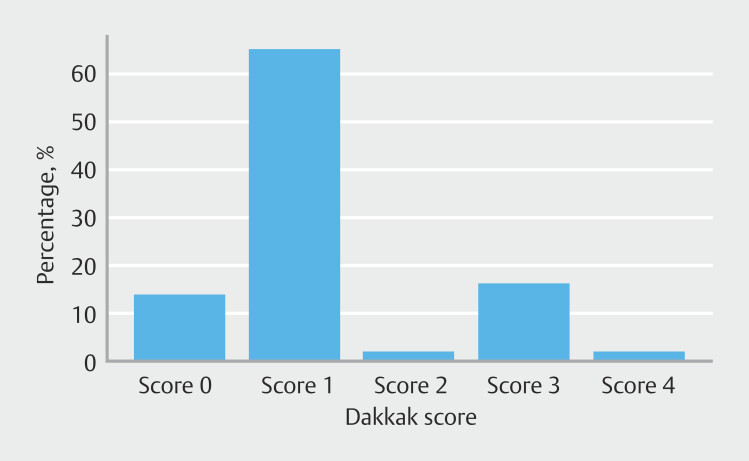
Distribution of Dakkak score in patients.

**Fig. 4:**
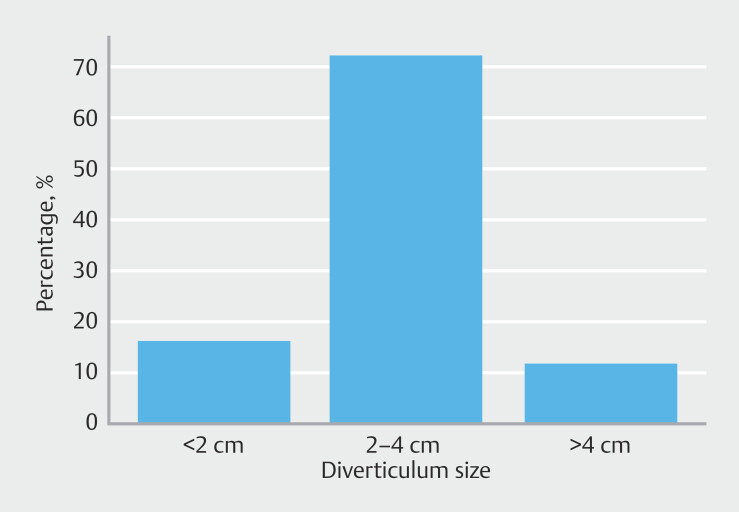
Distribution of Zenker’s diverticulum sizes.

Fig. 2 , Fig. 3 , and Fig. 4 are representations of symptoms, Dakkak Score, and diverticulum sizes.

Notably, there were no major complications such as obvious perforations or bleeding requiring reintervention, no infections, and no procedure-related deaths, except for presence of a piriform sinus wound in one of the patients, which was managed conservatively.

Contrary to ESGE recommendations, prophylactic antibiotics were administered to 41 of 43 patients (95.3%) with an average duration of 4 days.

Clips were used in 42 of 43 patients (97.7%) based on endoscopist practice or clinical need, such as bleeding or suspected perforation, consistent with the suggestion by ESGE.

Follow-up was conducted in 37 of 43 patients because the other patients were lost to follow-up.

Evaluation of symptoms was done on a case-by-case basis by each patient in a subjective manner. Each patient individually described improvement in their symptoms over time and provided this description in terms of a percentage. In addition to a subjective evaluation, an objective assessment was made in the patients, again using the Dakkak score to measure improvement in their symptoms.

Short-term follow-up of the procedure (1–3 months) showed major or complete improvement, with a Dakkak score of 0 in 92% of patients. In medium-term follow-up (3–6 months), major or complete improvement was observed in 94% of patients, also with a Dakkak score of 0. Long-term follow-up at 1 year showed major or complete improvement in 97% of cases.

**Fig. 5:**
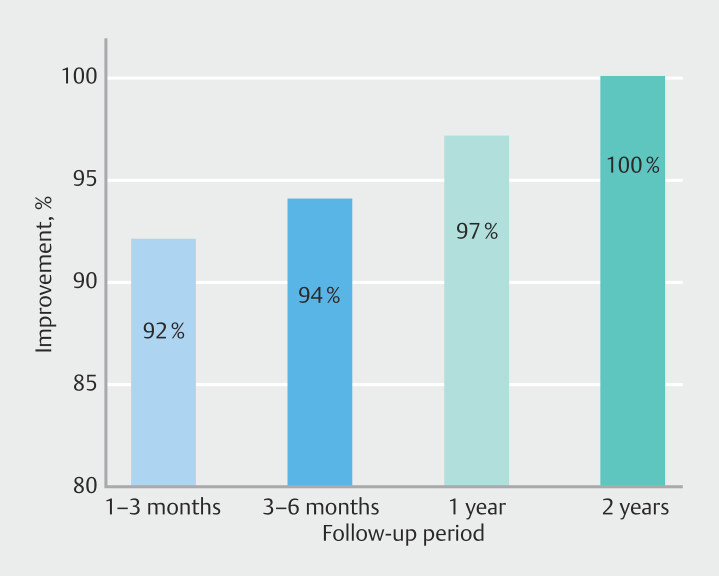
Follow-up improvement over time.

Among the 37 patients who were followed for 12 months, 24 (64.86%) were followed for 2 years with the same follow-up criteria. For this group of patients, stability in symptom improvement was observed with no recurrence of new symptoms and a Dakkak score of 0. Fig. 5 illustrates improvement over time during follow-up.

## Discussion

Treatment of ZD should be limited to symptomatic patients, with the primary goal being alleviation of symptoms and enhancement of patient quality of life. Two systematic reviews and meta-analyses, involving a total of 3,079 and 596 patients, have shown that open surgery yields higher clinical success rates (94%-96%) compared with the endoscopic approach (82%-87%) 10 11 . However, the endoscopic approach is associated with a significantly lower complication rate (11%-15% vs. 7%-9%).

ESGE recommends flexible endoscopic treatment as first-line therapy for symptomatic ZD.

Emergence of submucosal dissection and tunneling techniques like esophageal POEM has introduced new alternative strategies for ZD treatment through flexible endoscopy, with Z-POEM being the most promising, first described by Li et al. in 2016 12 . Yang et al. collected retrospective data on 75 patients treated with Z-POEM in 10 centers, reporting a technical success rate of 97%, a complication rate of 6.7% (1 bleeding and four perforations, all managed conservatively), a clinical success rate of 92%, and only one symptomatic recurrence after a median follow-up of 10 months 13 . Gaiani et al. consider diverticulotomy under digestive endoscopy of ZD by internal section of the diverticular septum as a safe technique with minimal morbidity, allowing significant and lasting improvement of symptoms in 80% of patients, recommending it as the first intervention for symptomatic ZD 14 .

Z-POEM is not recommended by ESGE except within the context of studies to define its role in managing ZD.

The American Society for Gastrointestinal Endoscopy considers flexible endoscopic approaches to be favorable for treating symptomatic ZD compared with traditional open surgical or rigid endoscopic alternatives.

In the study conducted by Al Ghamdi et al. 15 , symptoms recurred in 24 patients. Specifically, among the Z-POEM patients (15/102) with a mean follow-up of 282.04 days, symptoms recurred. In the group of patients who underwent flexible endoscopic septotomy (6/65) with a mean follow-up of 262 days, and in the group with rigid endoscopic septotomy (3/33) with a mean follow-up of 125 days, symptom recurrence was also observed.

AEs were reported in different percentages among rigid endoscopic septotomy (30.0%), Z-POEM (16.8%), and flexible endoscopic septotomy (2.3%).

In this international multicenter study, data suggest that all three techniques are effective in treatment of symptomatic ZD. However, Z-POEM exhibited a higher-than-expected recurrence rate. In contrast, flexible endoscopic septotomy had a shorter procedure time, achieved similar clinical success, and had fewer AEs when compared with Z-POEM and rigid endoscopic septotomy.

Repici et al. 16 asserted that flexible endoscopic septotomy (FES) represents a safe and effective treatment modality for patients with ZD. Despite a significant recurrence rate, endoscopic reintervention offers long-term relief of dysphagia. The procedure was successfully completed in all scheduled patients, with an early clinical success rate of 96.1%. AEs were reported in 3.5% of patients (9/256), with eight classified as mild to moderate, and no fatal events. However, one patient required surgery. Recurrences were observed in 31.3% of treated patients (80/256) after a mean of 9 ± 3 months, and notably, 95% of these recurrences were effectively treated by a second FES.

Goelder et al. 17 reported that during their study, no major complications such as perforation or mediastinitis occurred. However, five patients (9.6%) required a second treatment due to symptomatic recurrence after a mean of 7 months (ranging from 3 to 13 months). In addition, one patient underwent a third treatment (1.9%) without complications. Over a mean follow-up period of 16 months (ranging from 2–31 months), patients experienced a notable improvement in their dysphagia score, which dropped from an initial score of 2 (range 1–4) prior to treatment to 1 (range 0–4). Furthermore, symptoms such as odynophagia, regurgitation, and chronic cough were no longer reported in asymptomatic patients. These findings suggest that flexible endoscopic treatment of ZD using the SB knife and overtube is effective, safe, and provides lasting relief with a relatively low recurrence rate.

Building on this, Gölder et al. 18 proposed a unique "U-shape" technique, which offers several advantages, including providing a better overview during the procedure, preventing overly deep incisions, and reducing risk of perforation. Their preliminary data from 16 patients indicate that this innovative technique appears to be safe, with only one patient experiencing mild symptom recurrence. However, it is crucial to consider that published median follow-up duration of only 3 months may be too brief to draw definitive conclusions about the technique's long-term effectiveness. When compared with other studies of flexible endoscopic treatments for symptomatic ZD, these preliminary findings show similar results in terms of technical success, clinical success, and reintervention rates.

Shifting our focus to Nicolás González et al. 19 , their study explored use of the Clutch Cutter for endoscopic treatment of ZD, offering an easy, fast, safe, and efficient alternative for retreatment. Based on their findings, this technique presents a viable option for endoluminal, minimally invasive retreatment of ZD.

Rath et al. 20 believe that the Clutch Cutter (CC) allows for precise and deep incisions, leading to almost complete disappearance of the septum after a single treatment session. In their study conducted from 2015 to 2017, they reported a remarkable technical success rate of 100%. Six patients (3 females, 3 males) with symptomatic ZD underwent diverticulotomy using the CC, and complete diverticulotomy was achieved in a single session for all patients, with a mean duration of just 19 minutes. Importantly, no major complications, such as perforation or severe bleeding requiring reintervention, were observed during diverticulotomy with the CC. In contrast, mean procedure time with the needle-knife was significantly longer at 43 minutes, and a mean of 2.7 myotomy sessions were required for complete diverticulotomy.

Visrodia et al. 21 concluded that among novices performing ESD on an ex-vivo animal model, use of the CC knife was associated with a significantly lower rate of AEs without prolonging procedure time. This suggests that the Clutch Cutter may improve safety of ESD, particularly among learners.

The **Clutch Cutter** has emerged as a promising device for endoscopic treatment of Zenker’s diverticulum, although it has not yet been compared in randomized trials with other commonly used instruments such as the DualKnife, HookKnife, or SB Knife. One of the main advantages of the Clutch Cutter lies in its unique mechanical design, which combines a cutting blade with controlled traction on the muscle fibers. This allows for more precise and stable dissection, reducing risk of perforation and minimizing injury to surrounding tissue, as demonstrated by Rath et al. and González et al. 22 23 .

In contrast, conventional devices such as the DualKnife or HookKnife rely on a direct cutting mechanism, either with a blade or a snaring loop, which can sometimes limit procedure control. The Clutch Cutter’s traction capability enables better handling of tissue, leading to safer and more complete myotomy.

Clinical reports indicate that the Clutch Cutter is effective not only in primary interventions but also in retreatment of recurrent diverticula. Rath et al. reported successful primary endoscopic myotomy with minimal complications, whereas González et al. demonstrated that the device can be safely used for retreatment in cases of symptomatic recurrence, achieving effective myotomy and rapid recovery 22 23 .

Overall, the combined cutting and traction mechanism of the Clutch Cutter provides a significant technical advantage in the narrow and challenging anatomy of the esophagus, potentially reducing residual tissue and muscle injury, and improving patient outcomes.

Conventional devices such as the **HookKnife** and **DualKnife** rely on a direct cutting mechanism, which can limit endoscopist control over tissue manipulation, particularly in the narrow and delicate anatomy of the upper esophagus. The **HookKnife** , with its curved snaring tip, allows the endoscopist to grasp and cut muscle fibers selectively, which can improve precision compared with simple needle-knife techniques. Rouquette et al. reported that the HookKnife is both effective and safe for flexible endoscopic myotomy, achieving high rates of technical and clinical success while maintaining a low incidence of complications such as perforation or significant bleeding 24 .

Moreover, Mittal et al., in a large multicenter study, evaluated practice patterns and outcomes across multiple institutions using the HookKnife. Their results confirmed that Flexible endoscopic myotomy is an effective therapy for ZD, with a low rate of AEs. There was no significant difference in outcomes between traditional septotomy and a submucosal dissection approach, or with centers with higher volume, although clinical success was higher with the hook knife. 25 .

For the **DualKnife** and other submucosal dissection knives, which utilize a straight cutting blade, Laquière et al. demonstrated that these devices are effective for achieving complete myotomy. However, their use may be associated with longer procedure times and occasionally, multiple treatment sessions are required to fully divide the cricopharyngeal muscle fibers. Lack of a traction mechanism, compared with devices like the Clutch Cutter, can make tissue handling more challenging, potentially increasing risk of residual diverticular tissue or incomplete myotomy in less experienced hands 26 .

The **SB Knife** , particularly when used with an overtube, has also proven to be effective and safe, providing durable results with a relatively low recurrence rate. The overtube stabilizes the septum, while the SB Knife enables precise cutting and coagulation of cricopharyngeal muscle fibers, reducing risk of perforation and other complications. This combination makes the SB Knife a reliable alternative for endoscopic myotomy, especially in centers experienced with advanced endoscopic techniques 27 .

We hypothesize that the very low rate of recurrence or persistence of symptoms at 1 year and even up to 2 years in a subset of our population is attributable to complete sectioning of the cricopharyngeal muscle fibers, made possible and safe by the traction technique with dissecting scissors.

Similarly, absence of severe post-intervention complications, such as perforation or infection, is likely due to complete closure of the defect using through-the scope (TTS) clips, in contrast to the initially described technique, in which only one or two clips were placed at the base of the incision.

Furthermore, clips—whether delivered TTS or by other means—are widely used deep within the septotomy by a majority of endoscopists. However, despite their widespread use, there is currently no concrete evidence to suggest that application of clips significantly impacts AEs such as bleeding or perforation. As a result, the ESGE leaves the decision to use clips at the discretion of the operator.

These findings collectively emphasize the importance of considering various techniques and their outcomes in optimizing treatment of ZD.

Our study has several limitations: its retrospective, single-center nature, and the fact that the procedure was performed by a single, experienced operator in submucosal dissection, who has treated more than 70 diverticula throughout their career.

## Conclusions

Following several studies that demonstrated the success of cricopharyngeal myotomy using the Clutch Cutter, our study validates these findings in a larger cohort.

Myotomy with the Clutch Cutter was feasible in all our patients, achieving a technical success rate of 100%, with no AEs, particularly perforation, and no clinical recurrence of previous symptoms in 97% of patients at 1-year follow-up.

We believe that, in our series, the Clutch Cutter enabled precise and deep incisions, leading to almost complete disappearance of the septum after a single treatment session.

Currently, there are no randomized trials comparing different devices or techniques for ZD treatment. As a result, choice of technique typically depends on the physician's expertise and preference. We strongly believe that future randomized studies comparing different flexible endoscopic approaches for diverticulotomy are crucial to identify the most effective and safest technique for endoluminal therapy of ZD.
